# Supplementary material for: Are carbon emissions trading and green financial instruments synergistic? -Comprehensive quantitative research based on content analysis
Source: PLoS One. 2024 Mar 7;19(3):e0298601. doi: 10.1371/journal.pone.0298601 (PMC10919601; doi:10.1371/journal.pone.0298601)
Supplement: S1 Data — (ZIP) [file pone.0298601.s001.zip › Data/地级市Z_F工作报告.zip!/╜¡╦╒/┴1⁄4╘╞╕█╩╨/└┤╫╘╣1⁄2╓┌║┼í╛┬φ┐╦╩2╛▌═°í┐_2013.docx]

各位代表：现在，我代表市人民政府向大会作工作报告，请予审议，并请各位政协委员提出意见。

2012年工作回顾

刚刚过去的2012年，是我市发展史上不平凡的一年。在中共连云港市委的正确领导下，全市上下深入贯彻落实科学发展观，牢牢把握国家战略叠加的机遇，积极应对宏观经济下行、重大自然灾害侵袭等严峻挑战，统筹推进各项工作，保持了经济社会平稳较快发展的良好态势。预计实现地区生产总值1600亿元，同比增长13%左右；三次产业结构为13:48:39，二三产业比重较上年提高1.5个百分点；完成公共财政预算收入208.9亿元，增长16%；全社会固定资产投资、社会消费品零售总额分别增长23%、14.6%；居民消费价格涨幅和城镇登记失业率均低于控制性目标，较好完成了市人代会确定的年度目标任务。一年来的工作主要有以下几个方面：

一、加强组织谋划，经济发展稳中有进

深化思路促发展。把握又好又快工作导向，从抢抓战略机遇、实现跨越发展的全局出发，在港口、产业、城市、农村、民生、生态、创新、文化等方面形成一系列远近结合、重抓当前的操作性思路，制订完善具体措施，扎实推进各项建设和发展。围绕港口转型升级、综合发展、做大做强，同步推进港口能力建设、结构调整和功能完善；按照制造业出城、服务业进城的思路，拓展和优化城市产业发展空间框架，加快产业集聚发展、转型发展；突出规划引领，明确两新一老五组团布局，全面深化完善规划体系，迅速掀起城市建设热潮；坚持城乡统筹，推进新一轮乡镇区划调整，开展城乡统筹发展试点工作，加快推进农村改革，积极构建城乡一体化发展的体制机制；实行目标项目化、任务阶段化、工作责任化管理，加大组织力度，加快工作节奏，推动各项目标任务有效落实。

多措并举稳增长。加大经济工作组织力度，千方百计稳定存量、培植增量、扩大总量。狠抓项目带动，组织开展工业投资双强双促、项目集中开工等推进活动，500个重点项目完成年度投资923亿元。积极促进需求拉动，及时研究制定对策，进一步刺激消费、扶持外贸发展。强化要素保障，抓好土地、资金、用工和煤电油气运调度。多元拓展融资渠道，着力增强金融支撑，全市各项贷款余额1286.7亿元，新增中小微企业贷款90亿元、涉农贷款83.7亿元。新增上市企业2家，企业直接融资总额113.8亿元。落实财政奖励、挂钩帮扶等措施，清理规范涉企收费，支持企业扩销挖潜、减负增效，累计减免退税30亿元。全市实体经济总体保持了效益提升、稳定发展的良好势头。规模以上工业产销率97.8%，经济效益综合指数较上年提高29个百分点。

二、重抓港口建设，沿海开发成效明显

一体两翼组合大港加速形成。连云港区25万吨级航道正式通航，30万吨级矿石码头批对外开放，氧化铝、散化肥码头建成试投产。徐圩港区10万吨级航道和2个10万吨级泊位加快建设，东防波堤开工建设。赣榆港区5万吨级航道建成通航，实现开港运营。灌河港区灌河口拦门沙治理一期工程完工，2万吨级航道通航，海河联运区和燕尾作业区一批万吨级泊位加快建设。主体港区转型升级实质性启动，港容港貌整治出新初显成效，散杂货实施标准化堆放，船用岸电技术上升为国家标准，获批建设国家海铁联运物联网应用示范工程，成为全国低碳试点港和科技示范港。

集疏运体系逐步完善。旗台作业区铁路专用线主体完工，矿石带式输送机工程有序推进。北疏港公路、徐圩港前大道、徐新公路、242省道、310国道加快建设，临海高等级公路埒子口至青口段简易通车。全长120公里的港产城联动发展交通大动脉海滨大道开工建设。盐河航道建成通航。连盐铁路建设前期工作积极推进。新开国际集装箱航线8条，3条铁路班列纳入全国客车化运营，率先开通经霍尔果斯口岸出境的集装箱班列。港口完成货物吞吐量1.85亿吨、集装箱502万标箱，客运量12万人次，海铁联运量居全国首位。

重大事项重点项目取得积极进展。国家东中西区域合作示范区建设全面展开，徐圩新区一区七园发展格局初步形成，示范区重大功能平台建设加快推进，出口加工区二期封关运作。列入省、市沿海开发三年计划的180个重点项目如期推进，累计完成投资1269亿元。新机场建设、空港开放列入国家专项规划。炼化一体化项目取得实质性进展，神华能源基地等一批重大产业项目签约。田湾核电二期工程开工建设，新电公司首台百万千瓦机组并网发电，珠江钢管、益海盐化、华乐合金等重点产业项目建成投产。徐圩新区送水工程、三洋港挡潮闸等重点水利工程完工。一批输变电工程投入运行，我市电网升级为独立的分区电网。

三、加快转型升级，产业发展增量提质

工业经济加快壮大。大力实施双千双百工程，推动产业集中集聚集约发展，规模以上工业增加值增长17.4%、利税增长31%。主导产业和特色产业较快发展，石化、冶金等临港产业实现产值1650亿元，三新一高产业实现产值1600亿元。新医药产业增势强劲，产值增长41.3%，硅资源等5个特色产业和新海石化、益海粮油等4家企业销售收入过百亿。产业集中集约效应逐步显现，开工建设亿元以上工业项目144个，100个新增长点项目形成产值400亿元。市开发区率先建成千亿园区，六大园区销售增幅高于规模以上工业3.5个百分点，园区工业增加值占规模以上工业70%。14个中小企业园、20个工业集中区加快建设。扎实推进节能减排工作，完成省定节能减排目标任务。

现代服务业加快发展。突出抓好集聚区和重点项目建设，加快打造26个现代服务业集聚区，52个服务业重点项目完成投资93.9亿元，服务业增加值增长12.4%。新兴服务业不断拓展，酷歌动漫产业园、省级服务外包示范区加快建设，保税物流中心进出货值14亿美元。金融服务体系不断完善，太仓农商行新浦支行等5家金融机构开业，东方农商行、灌云农商行挂牌运营。民航增开至香港等3条航线，旅客吞吐量突破50万人次。积极构建大云台山旅游格局，花果山5A级景区创建和国家级旅游标准化试点扎实推进。新增国家级景区11个，大伊山景区、羽泉景区成为4A级景区，东海温泉获批省级旅游度假区。游客接待量、旅游总收入分别增长15%、20%。传统服务业稳步提升，兴隆商贸广场等一批商贸项目建成营业。

农业现代化加快推进。克服严重自然灾害影响，粮食生产实现十连增。高效农业规模不断扩大，新增高效设施农业10万亩、高效渔业10.3万亩，建成千亩连片基地81个、万亩连片基地8个。燕尾港获批为国家一级渔港。农业产业化水平稳步提升，新增省级农业产业园区2家、市级以上龙头企业42个，农业适度规模经营比重65%。农业标准化、外向化水平不断提高，新增无公害、绿色、有机农产品485个，国家地理标志保护农产品达7个。灌南县获批省级食用菌农业标准化示范县，花果山蔬菜和赣榆泥鳅出口农产品质量安全示范区通过国家级认定。完成农产品出口4.1亿美元，增长10%。县域经济加快发展，23个乡镇财政收入过亿元，赣榆、东海两县达到全面小康标准，赣榆县进入全国百强县行列。

四、实施组团开发，城乡建设统筹推进

城市形象快速提升。新一轮城市建设框架全面拉开。老城区加快改造升级，完成旧城改造72万平方米。全长34公里的BRT一号线建成运营，瀛洲路等7条道路开展整治，城市主干道面貌焕然一新。连云新城建设全面加快，完成投资60亿元，三级路网基本形成，海州湾国际会议中心、美食水岸等一批重点项目有序推进。新海新区33条道路基本建成，市体育中心一场三馆全面完工，市公共卫生中心、广播影视城、花果山国际酒店、福利中心主体封顶，科技创业城、景观湖公园开工建设。市开发区创智街区建设加快，徐圩新区城市功能配套快速完善，滨河组团、盐河组团、国际商务中心组团有序推进。市区建成区面积突破140平方公里、人口超过106万。全市城市化率55%。

城市功能加快完善。实施市政道路桥梁工程120项，金海大道、秦东门大桥建成通车。完成排淡河治理工程，新改建雨水管网100公里，南城污水处理厂和沙板桥污水泵站建成运行，城市污水处理率82%。城市饮用水安全保障工程扎实推进，蔷薇河下游段完成清淤，流域环境综合整治取得阶段性成果，自来水深度处理一期工程建成运行。实施东盐河景观绿化、新浦公园改造等重点园林绿化项目111项，新增绿地380公顷。苏宁广场、振兴时代广场等城市综合体加快建设。城市精细化管理进一步强化，四城同创积极开展，获批为国家园林城市。

城乡建设力度加大。四县实施城建项目500项，完成投资300亿元。统筹城乡发展镇村规划工作全面启动，完成首批11个试点乡镇、19个试点村规划编制任务。实施215个新农村建设重点项目，灌南县汤沟镇、东海县白塔埠新镇区和赣榆县柘汪镇王坊新社区等一批新型镇村建设初见成效。惠民实事工程扎实推进，新改建农村公路462公里，行政村客运班车通达率99.7%，农家店村覆盖率97%。美好城乡建设行动深入开展，完成1285个村庄环境综合整治任务，新增20个省三星级康居示范村。四县全部建成国家级生态示范区，赣榆县青口镇获批国家级生态乡镇。全市林木覆盖率26.6%。秋季秸秆实现全面禁烧，秸秆综合利用率86%。

五、强化创新驱动，发展活力得到增强

创新能力持续提高。新认定国家级高新技术企业30家、企业技术中心1家，创建两化融合示范试点企业25家，大中型工业企业研发机构实现全覆盖。新增中国驰名商标6件。中复神鹰T700高性能碳纤维实现规模化生产，恒瑞医药成为全国首家制剂产品进入美国和欧盟市场的企业。重大研发平台建设取得新进展，中科院能源动力研究中心开展科技研发。创新资源加快集聚，争取国家重大科技专项8项，实施星火计划项目70项。全社会研发支出占地区生产总值1.3%。高新技术产业完成产值1300亿元，占规模以上工业37%。组织实施三百引才等人才集聚工程，引进高层次创业创新人才900人、领军人才48人，2人入选国家千人计划、10人入选省双创计划，人才支撑不断强化。

重点改革加快推进。创新体制机制，增强发展活力，成立新城开发等指挥机构，组建市旅游发展集团。投融资体制改革不断深入，国有资产公司专业化发展步伐加快。价格改革逐步深化，居民阶梯式电价全面实行。农村产权、土地、户籍、金融、就业和社保制度改革统筹推进，东海县建成全国首家县级农村产权交易所，柘汪镇获批为国家第三批小城镇综合改革试点镇。文化体制改革扎实推进。医药卫生体制改革不断深化，公立医院运行机制逐步完善，村卫生室基本药物制度实施率100%。

开放型经济工作得到加强。以更高的水平请进来，高标准举办连云港之夏旅游节暨沿海开发经贸洽谈会、首届东中西区域合作论坛暨西游记文化节等经贸活动，吸引16个国家和地区的400多家客商、37家世界500强企业来连投资洽谈。以更大的步伐走出去，设立5个境外招商机构，在韩国、新加坡、香港、台湾等国家和地区高质量举办大型经贸招商活动，港口、物流、旅游等49个项目成功签约，71个重大招商引资项目有序推进。全市直接利用外资8.1亿美元，增长32.6%。内联客方到位资金611亿元，增长15.9%。完成外贸进出口78亿美元，增长13%。国际服务贸易达到13.2亿美元，增长62%。支持企业开展境外投资，新核准境外投资项目5个，对外直接投资1.1亿美元。

六、着力保障民生，人民生活持续改善

城乡居民收入稳步提高。认真落实十大民生工程，民生领域支出占公共财政预算支出72%。推动就业创业富民，开展创业及各类职业技术培训32万人次，实现自主创业并带动就业1.1万人。加快15分钟公共就业服务圈建设，组织开展创业贷款助推和充分就业双创行动，积极促进高校毕业生、农村劳动力和城镇困难人员就业。新增城镇就业8.1万人、农村劳动力转移就业1万人。实施脱贫奔小康工程，实现10万低收入人口脱贫、40个经济薄弱村达到省定脱贫目标。城市居民人均可支配收入达到24840元，农民人均纯收入达到9700元，分别增长14.5%、15%。

社会保障水平不断提升。统一城乡居民社会养老保险制度，职工五项社会保险参保人数平均新增3万人。企业退休人员养老金人均每月增加173元。城镇职工和居民医保住院医疗费用报销比例分别为81%和70%。新农合实现应保尽保，支付方式改革经验在全国推广，重大疾病救治病种增至20个。稳步提高城乡居民低保标准和医疗救助比例，发放各类救助资金3.1亿元。新建平价商店33家、菜篮子生产基地16个，改造城区农贸市场12个。采取集中公开收购、发放货币补贴等方式，拓宽保障房筹集渠道。全市开工建设各类保障性住房10874套，竣工7390套，住房困难保障标准扩大到人均18平方米。落实惠老助老政策，为10万名80岁以上老人按时足额发放尊老金。慈善募捐和红十字救助工作得到加强，老龄和残疾人康复事业加快发展。

社会事业全面进步。各类教育发展水平不断提高，省优质幼儿园比例达66%，公办普通高中和中等职业教育全部建成省三星级以上学校，基本实现教育现代化。中小学校舍安全工程三年任务基本完成。连云港社区大学成立。义务教育免费政策和贫困生资助制度全面落实，1.5万名进城务工人员子女顺利入学。公共卫生服务体系不断完善，省级示范基层医疗卫生机构增至32家，基本公共卫生服务项目增加到10类41项。在全省率先建成区域卫生信息平台，发放居民健康卡6万张。市食品安全快速检测中心建成运行，药品不良反应监测实现全覆盖。统筹推进城乡文化发展，繁荣文艺创作生产，举办惠民展演活动400多场，有线电视村村通工程和新闻出版工作有效推进。加快城市10分钟体育健身圈建设，全民健身和竞技体育取得新进展，赣榆县获评中国拔河之乡。人口管理服务措施不断加强，妇女儿童、民族宗教、气象、防震减灾、档案、地方志、关心下一代和老区开发事业取得新进步。

社会管理得到加强。组织实施社会管理创新工程，强化基层基础建设，所有县区和90%以上乡镇街道建成社会管理服务中心，新增全国民主法治示范村2个，开通互联社区网，东海县122民生热线经验在全国推广。深入开展三解三促、四项排查活动，健全矛盾纠纷大调处机制，狠抓信访问题源头化解，扎实做好安全生产工作。巩固平安连云港、法治连云港建设成果，建立侦查、研判、巡防三项合成警务机制，有效维护社会治安，公众安全感和法治建设群众满意度得到提升。全面推进行政审批服务三集中四到位，进一步提高行政效能。加快惩防体系建设，建成电子化招投标系统，反腐倡廉工作得到加强。大力推进公民道德建设，广泛开展群众性精神文明创建活动，雷锋车组成为全国重大典型。国防动员、民兵预备役和优抚安置工作取得新成绩。

各位代表，回顾过去一年，我们经受了宏观环境复杂多变的严峻考验，夺取了抗击历史罕见的干旱、暴雨、台风灾害的重大胜利，展现了大力度、快节奏、高质量推进城市建设的崭新风貌，更增强了应对困难、推动发展的能力，积累了创新思路、破解难题的经验，锤炼了顽强拼搏、连续奋战的作风，坚定了迎难而上、敢打必胜的信心。所有这些，归功于市委的正确领导，归功于市人大、市政协的监督支持，归功于社会各界的共同奋斗。在此，我代表市人民政府，向全市人民，向人大代表、政协委员和离退休老同志，向各民主党派、工商联、人民团体和无党派人士，向驻连部队、武警官兵、公安干警和驻连部省属单位，向热心支持连云港发展的海内外人士，表示衷心的感谢！

在肯定成绩的同时，我们也清醒地看到，我市经济社会发展中还存在不少困难和问题：规模以上企业数量较少，产业实力和竞争力不强，重大项目还需要加快突破；资源环境约束加剧，要素瓶颈制约还没有很好地破解，转型升级的步伐需要进一步加快；城市建设管理水平仍需加快提升，环境保护和生态建设需要进一步加大工作力度；小康社会建设存在不少薄弱环节，统筹城乡发展、改善群众生活还有大量工作要做；投资发展软环境还存在一些突出问题，机关作风建设需要进一步加强。对此，我们一定高度重视，不回避矛盾，不掩盖问题，深入分析原因，积极采取有效措施切实加以解决。

2013年主要工作任务

2013年是全面贯彻落实党的十八大精神的开局之年，是实施十二五规划承前启后的重要一年，也是全市深入推进沿海开发、全面建成小康社会的关键之年。综观当前形势，世界经济已经进入深度转型调整期，低速增长态势还将延续，宏观环境依然复杂严峻，不确定因素仍然较多，风险和挑战不可低估。但是，我们更要看到，我国仍处于可以大有作为的重要战略机遇期，经济社会发展基本面长期向好的态势没有变。特别是党的十八大对新的时代条件下推进中国特色社会主义事业作出了全面部署，为我们紧紧抓住用好重要战略机遇期，不断开创科学发展新局面指明了方向。当前，我市国家战略聚焦的机遇效应正在加快释放，港产城联动发展的新格局正在加快形成，广大干部群众的思想观念和能力作风正在深刻变化，推动连云港崛起跨越的有利条件和积极因素正在不断累积。全市上下必须进一步坚定后发先至的实践自信、路径自信和前景自信，进一步强化加快跨越的思想自觉、责任自觉和行动自觉，咬定目标，抢抓机遇，不懈奋斗，全力以赴把连云港崛起振兴的事业推向前进。

根据市委十一届五次全会的部署，今年政府工作的总体要求是：在党的十八大精神指引下，坚持稳中求进、好中求快，项目推动、重点突破，加快全面小康建设进程，加快转型升级步伐，加快港产城联动发展，加快县域经济发展，加快提升中心城市现代化和国际化水平，着力在港口建设及港容港貌、重大产业项目及特色产业和园区建设等十个方面实现突破，着力推进事关群众切身利益的十大民生工程，推动全市科学发展、跨越发展取得更大成效。主要预期目标是：地区生产总值增长13%左右；公共财政预算收入增长18%左右；全社会固定资产投资增长23%；直接利用外资9亿美元；内联客方到位资金710亿元；社会消费品零售总额增长15%；城市居民人均可支配收入增长13%，农民人均纯收入增长13%；城镇登记失业率控制在3.6%以内；居民消费价格涨幅不高于省控标准；节能减排完成省定目标。

新的一年，围绕全面建成小康社会，我们将强化跟踪分析、动态监测，实施分类指导、重点攻坚，对消费水平提升、产业结构调整等4个难点指标强力组织推进，对已经达标的21个指标继续巩固提高，把各项指标分解为具体工作任务，融入到经济社会发展当中，逐项落实责任，落实措施，确保按序时进度达到目标要求。在此基础上，积极推动赣榆、东海做好达标验收和巩固提升工作，支持灌云、灌南加速小康建设进程，为明年全市以县为单位全面建成小康社会奠定坚实基础。

围绕上述目标和要求，着力做好八个方面工作：

一、奋力开拓沿海开发新阶段

全面启动沿海开发五年推进计划第二阶段建设，加快做强港口引擎，优化硬件支撑，增强承载能力，在港产城联动发展上取得突破。

加快港口综合发展。以港口功能提升和港容港貌出新为重点，完成港口建设投资35亿元，全面形成一体两翼港口发展格局。连云港区建成30万吨级航道一期工程，开工建设二期工程，加快旗台作业区液体化工等泊位建设，建成新客运码头和客运站。徐圩港区进一步推进航道、防波堤、码头和配套设施建设，实现开港运营。赣榆港区实现正常运营，开工建设10万吨级航道，继续实施码头和防波堤工程。加快集疏运体系建设，建成公路中心货运站和内河港中云作业区一期工程，推进航线开辟、班列增设和物流场站建设，大力拓展航运市场、货源市场。继续推动港容港貌出新和货种布局结构调整，新建改造一批港口服务设施，完善口岸公共信息平台功能，推广应用船用岸电技术，建立港口资源循环利用系统。积极培育加工贸易、航运服务等港口经济，推动港产城联动发展取得新进展。完成港口货物吞吐量2亿吨、集装箱运量540万标箱。

加快重大基础设施建设。以完善体系、增强支撑为重点，实施交通、水利、能源等49项重点基础设施项目，完成投资59亿元。开工建设204、310国道市区段，启动建设235省道改线工程，实现242省道全线通车。加快北疏港隧道和墟沟互通工程建设，建成临海高等级公路和徐圩港区港前大道，完成徐新公路路基工程和大港路中期改造。推进连淮铁路、连徐客专及两翼港区铁路支线前期工作，开工建设连盐铁路。完成新机场选址和项目立项前期工作，力争开工建设。全面完成灌河航道整治，实施连云新城段海堤达标和徐圩新区防洪除涝等重点工程。加快田湾核电二期工程建设，完成输变电工程11项。

加快国家东中西区域合作示范区建设。以五大功能区为重点，着力打造区域合作服务平台。突出抓好先导区建设，加快热电、污水处理等基础设施建设进度，开工建设27条道路，建成40公里水电管网。争取建立示范区建设部省联席会议制度，进一步完善示范区共建共享和产业转移政策，加快构建出口产品生产基地等功能平台，积极引进中西部地区参与示范区建设。全力突破重大政策事项，推动国家和省支持政策发挥效应。力争空港和赣榆、徐圩港区口岸正式开放，积极争取设立综合保税区，推动组建江苏沿海发展银行总部，做好招商银行连云港分行设立工作。

二、积极增创转型升级新优势

推动经济增长与转型发展互促并进，在重大产业项目、特色产业和园区建设、创新发展及平台建设上取得突破，迅速壮大产业规模、提升发展质量。

千方百计促进经济稳定增长。坚持以项目为纲，有效发挥投资对稳增长、调结构、惠民生的关键作用，组织实施500个年度重点投资项目，开展难题会办等项目促进活动，持续扩大有效投入，完成全社会固定资产投资1910亿元、工业投资1150亿元。完善落实扩大消费的政策，营造安全放心的消费环境，大力培育文化、信息等热点，增强消费对经济增长的拉动力。强化存量挖潜和增量培育，落实企业帮扶措施，切实保障要素供给，着力推动80家行业代表性企业稳定发展，工业20强企业产值增长20%以上，新增规模以上企业100家。加大金融服务实体经济力度，推进金融产品和服务创新，开展金融服务日等银企对接活动，设立中小企业融资超市，健全融资担保体系，积极拓展直接融资。争取金融机构贷款余额增幅高于全省平均水平，中小微企业贷款增幅高于全市贷款增幅，新增企业直接融资30亿元。

加快工业经济扩量提质。以临港产业突破、新兴产业发展和特色产业培育为重点，实施投资过亿元的重点开工和竣工项目各100项，投资过3000万元以上技术改造项目100项，年销售过5000万元新增长点项目100项，完成工业总产值4000亿元，规模以上工业增加值增长17%。加快推进醇基多联产、宝通镍业等临港产业项目，力争开工建设炼化一体化等基地型大项目。石化、冶金产业销售收入分别突破800亿元、600亿元。大力实施高新技术产业双提升计划，开工建设正道新能源汽车等项目，加快奥神环保纤维等项目建设。推动光伏产品终端应用，引导光伏产业实施结构调整。大力发展海洋生物医药、海洋工程装备、海水综合利用等海洋产业。新医药、新材料、新能源三新产业销售收入达到1100亿元，高端装备制造产业销售收入突破千亿。深入实施双千双百工程，着力培育重点产业链和大型企业集团，促进产业特色化、规模化、集聚化发展。六大园区年销售总额达到3900亿元，百亿特色产业达到8个，产值过50亿元的企业达到20家，力争新海石化产值超过200亿元。

推动服务业优化升级。围绕企业生产经营链和居民行为需求链，重点培育15个服务业集聚区，50个重点服务业项目完成投资130亿元。服务业增加值增长13%。大力发展现代物流、商务服务、研发设计等生产性服务业，加快临港物流园区、工业邻里中心、服务外包基地等载体建设，促进企业快速集聚。积极发展商贸流通、旅游休闲等生活性服务业，建成一批大型商贸设施，培育做大特色商业功能区。继续推进万村千乡市场工程和社区商业双进工程，健全城乡流通网络。提升旅游景区开发建设水平，推进花果山国家标准化景区试点和5A级景区创建，建成海州湾国际会议中心、花果山国际酒店等一批旅游配套设施，促进旅游产业化发展、品牌化经营。游客接待量、旅游总收入分别增长12%和15%。实施服务业创新百企示范工程，积极培育大型现代服务业企业，推动物联网应用示范工程、网络与信息安全监测中心、农村综合信息服务平台及城市智慧小区建设，加快发展电子商务、网络购物等新型业态。强化房地产市场调控和引导，促进房地产市场和住房保障协调健康发展，努力满足不同人群的住房需求。

着力增强创新发展能力。突出人才第一资源的创新引领作用，加速集聚创新创业人才，引进高级管理人才100人、高端专业人才100人、复合型高层次创业创新领军人才30人。新增国家级博士后工作站1家、省级博士后创新基地2家。启动企业创新能力提升计划，深化政产学研金合作，促进科技与金融结合，加大全社会研发投入力度。培育高新技术企业30家，实施国家重大科技专项20项、重大科技成果转化项目10项。专利申请量、授权量分别达3800件和1800件。加快重大创新平台建设，积极申报国家重大科技基础设施项目，建成中科院能源动力研究中心技术平台、省海洋资源利用功能材料重点实验室。组建全国电子封装材料标准化分技术委员会。完善创新服务体系，加快市开发区五园五中心建设，建成医药公共服务平台，科技创业城一期实现运营。全市新增创新创业载体面积60万平方米。推动省级高新区升格为国家级高新区，支持东海开发区、赣榆海洋经济开发区创建省级高新区。实施质量强市和品牌兴市战略，推进企业质量信用评价，争创省名牌产品10个，采用国际标准30项。

三、全面塑造海滨城市新形象

围绕现代化国际海港中心城市目标，加快完善城市功能，着力提升城市品位，在新城建设、老城改造和环境整治上取得突破。

高标准推进组团开发。制定并全面实施城市规划、建设、管理导则，加强精细管理，严格控制用地性质、容积率、停车位等强制性指标。连云新城加快打造金融商务、休闲度假核心区，完成投资120亿元，重点实施85个项目，加快建设时尚文化街区、企业总部功能区和一批公共服务项目。新海新区全力打造科技、文化、旅游等功能板块，完成投资120亿元，重点实施65个项目，开工建设新区医院，建成科技馆、海洋馆。市开发区继续抓好创智街区建设，沿花果山大道高标准建设一批地标建筑。徐圩新区加快建设大陆桥资源采购中心、高级人才公寓等重点项目。继续推进新浦滨河组团、海州盐河组团、连云国际商务中心组团开发，努力打造精品工程。推动建筑市场健康发展，实现建筑业增加值120亿元。

全方位完善城市功能。重点实施63个城建项目，完成投资183亿元。进一步优化城区路网，加快BRT支线建设，实现海滨大道全线贯通。大力构建城市饮用水保障体系，开工建设取水口上移工程，同步推进蔷薇湖生态净化工程。加强水、电、气、通信等设施建设，完成第三水厂扩建，新增供水、污水和燃气管网各20公里。新增人防工程13万平方米。组织实施花果山截洪沟、大浦河下游拓浚、临洪西泵站改造等城市防洪排涝重点工程，努力提升城区防洪水平。推进市区热源点规划整合，加快完善供热供暖设施。快速完善城市公共配套，开工建设新苏天地等城市综合体，加快建设市开发区工业展览中心等一批商务服务设施。

大力度提升城市品质。强力推进老城区拆迁改造，重点实施幸福村、外仓街等90个旧城改造项目，年内完成房屋征收面积450万平方米，其中市区完成210万平方米，四县完成240万平方米。大力改善老城区道路、管网、卫生、绿化等配套设施条件，实施14条主次干道和30条背街小巷整治出新，美化亮化城区面积20万平方米。完成花果山景区出入口综合整治，推进海州古城、南城古镇、连云古镇保护与修复建设。启动西盐河、玉带河清淤工程。开工建设动物园，实施苍梧绿园升级改造等园林绿化工程，新增城市绿地300公顷。规范和加强住宅小区物业管理，切实提升服务水平。扎实推进四城同创，加大违建查处力度，健全城市管理快速反应和长效管理机制。大力整治交通秩序，切实解决渣土抛洒等影响市容环境的突出问题。

四、着力开创改革开放新局面

坚持以改革增动力、以开放增活力，在重点领域改革和外资招引上取得突破。

深化重点领域和关键环节改革。落实国家推进财税、收入分配、户籍、价格等改革的系列政策。稳步推进医药卫生体制改革，巩固完善基本药物制度，加快公立医院改革步伐，健全基层医疗卫生机构运行机制。深化文化体制改革，推进文化资源整合，组建广电集团、报业集团、演艺集团。加快国有资产管理体制改革，整合优化国有资本经营公司，增强资本运作能力。严格落实粮食最低收购价政策和涉农价费公示制度，有序推进农村产权、土地、税费等制度改革，创新农业农村投融资政策，积极开展一权一房抵（质）押贷款试点工作，扩大政策性农业保险试点范围。稳步推动资源性产品价格改革。加快提升对外开放水平。把招商引资工作摆在更加突出的位置，发挥县区和开发园区主阵地作用，进一步创新体制，充实队伍，着力抓好66个重大招商项目的协调服务。完善园区服务配套功能，推动开发园区转型升级，推进省级开发区申报扩区。瞄准日韩港台新等重点国家和地区，主动对接世界500强企业、大型央企、长三角地区和中西部重点产业，有针对性地推进项目洽谈对接。高质量办好走出去、请进来活动，提高组织化程度，形成强大的招商攻势，确保招商引资工作取得明显突破。积极转变外贸增长方式，重点培育机电产品、特色农产品和科技兴贸出口基地，突出做好30重点外贸企业、100家出口新增长点企业的服务，创建省级以上出口品牌3个，完成外贸进出口80亿美元以上。支持优势企业参与国际合作，建立海外研发和生产基地。继续加强外事、侨务、港澳、对台事务等工作。

五、扎实迈出统筹发展新步伐

推进城乡经济社会和资源环境协调发展，在城乡一体化建设上取得突破，着力打造美丽港城、生态家园。

大力推进新型城镇化。推动四县县城进一步完善功能、彰显特色，稳步实施乡镇区划调整、行政村撤并和自然村整合，加快优化城镇体系。扎实推进新镇区、新社区、新农居建设，配套完善生产生活服务设施，增强重点镇辐射带动功能，有序推进农业转移人口市民化，确保26个城乡统筹试点乡镇、69个试点村建设取得明显成效。加强农村基础设施建设，改扩建农村公路150公里，完成20万农村居民饮水健康工程。推进村庄环境综合整治，完成2024个村庄环境整治任务，启动50个新农村示范村建设。提升农村公共服务水平，促进城乡文化、教育、卫生、人口等服务体系均衡发展。推进扩权强镇，推动乡镇经济壮大规模、培育特色，加快增强县域经济实力。

大力推进农业现代化。开展农田水利设施建设年活动，实施区域性河道、中小河流治理、水闸泵站改造等重点工程。疏浚县乡河道1038公里、村庄河塘2869条，改善农田灌溉面积39万亩。稳定粮食生产，强化农业综合开发，提升农业机械化水平，新建高标准农田20万亩。加快发展现代高效农业，新增高效设施农业10万亩、高效设施渔业2万亩、优质林果茶基地3万亩，着力培育畜禽、蔬菜食用菌、林果花卉、浅海域渔业4个百亿产业，每个县建设1个规模化现代农业示范园区，每个乡镇建设1个特色农业园区。深入实施农业产业化战略，加快裕灌农产品加工产业园、雨润生猪养殖等重点项目建设，培育新型农业经营主体，延伸产业链条，提高产业组织化程度。新增专业合作社200家、农产品加工企业30家。完善农业科技创新体系，实现省级以上农业园区科技服务超市全覆盖。加强动植物疫病防控和农产品质量安全工作，积极推进无公害、绿色、有机农产品认证和原产地认定。加快大型农产品批发市场建设，构建农副产品三级流通网络，促进农超、农贸和农企对接。农产品出口额增长

10%。

大力推进生态文明建设。落实最严格的环境保护和水资源管理制度，合理开发水资源，科学开发海域海岛资源，节约集约利用土地资源。狠抓突出环境问题治理，积极控制工业点源和农业面源污染，着力整治工业园区和化工集中区环境。加大环保投入，推进城乡垃圾统筹处理，开工建设20个乡镇污水处理厂。强力推进秸秆禁烧和综合利用，着力健全长效管理机制，确保取得更大成效。严格落实节能减排目标责任制，强化重点用能单位目标考核和重点排污企业监管，实施节能重点技改项目30项、减排重点工程20项。积极发展循环经济、低碳经济和节能环保产业，推动省级以上开发区创建生态工业园。广泛开展低碳全民行动，深入推进公共机构节能工作。切实加强生态建设，创建花果山国家级森林公园和国家级地质公园，强化临洪湿地生态保护，推进海洋生态修复工程，实施市区开山采石塘口综合整治。建成8个国家级生态乡镇，新增绿化造林15万亩，林木覆盖率达27.1%。

六、加快推进社会事业新发展

顺应经济社会转型要求，加快发展社会事业，在改善公共服务供给上取得突破，使公共产品更多更好惠及广大群众。

扎实推进教育和卫生事业发展。坚持教育优先发展，启动建设更高水平教育现代化，推进学前教育优质普惠发展、义务教育优质均衡发展、普通高中优质多元发展、特殊教育健康持续发展。继续实施中小学校舍安全工程，新建和改造校舍22万平方米。开工建设师专二附小新校区、海宁中学滨河校区等重点工程，建成新海实验中学新海新区分校。推进职业教育规模特色发展。启动广播电视大学搬迁工程，支持淮海工学院硕士点建设，加快提升地方院校办学层次。优化配置教育资源，提高家庭经济困难学生资助水平，保证进城务工人员子女平等接受教育。健全农村三级医疗卫生服务网络和城市社区卫生服务体系，拓展区域卫生信息平台，深化乡村卫生一体化管理，实施全科医生团队与家庭医生制度。加强疾控机构达标建设，落实公共卫生服务项目，提高重大疾病防控水平。市传染病医院创成三级专科医院。广泛开展爱国卫生运动，启动健康港城促进行动。

繁荣发展文化事业和文化产业。加强重大公共文化工程和文化项目建设，提升文化服务供给能力。开工建设新海新区文化艺术城、市艺术学校新校区。继续办好连云港之夏、西游记文化节两大节庆活动。加强文艺创作生产，扩大文化品牌效应。实施文化惠民工程，广泛开展群众性文化活动，推进有线数字电视户户通，促进公共文化服务设施在免费开放的基础上完善功能、提升水平。加强文化遗产保护传承，保障文化市场安全有序。加快建设城市10分钟体育健身圈，深入开展全民健身运动，做好十二届全运会参赛组织工作。加大对各类文化企业、重点产业项目和新型文化业态的扶持力度，进一步提高文化产业规模化、集约化、专业化发展水平。

统筹推进各项社会事业全面发展。加强人口与家庭公共服务体系建设，开展计生幸福家庭创建活动。全市免费婚检率达70%以上，免费孕前优生健康检查实现全覆盖。稳定适度低生育水平，加强出生人口性别比综合治理，促进人口长期均衡发展。优化妇女儿童发展环境，积极解决流动人口和弱势妇女儿童实际困难，推进婴幼儿早期发展指导中心建设，妇女儿童之家建成率达100%。开工建设老干部活动中心，推进市档案馆新馆建设。加快市社会福利中心、民政公共服务中心、残疾人综合服务中心建设，大力发展慈善事业。做好第三次经济普查工作。加强国防动员、双拥共建和民兵预备役工作，推动民族宗教、地方志、老区开发、关心下一代和红十字事业再上新台阶。

七、大力推动民生质量新改善

坚持立足当前办实事、着眼根本建体系，深入实施民生幸福工程，在富民惠民上取得突破。

加快实施居民收入倍增计划。把充分就业、勤劳创业、发展实业作为增收的主渠道，完善和落实促进就业创业的扶持政策，努力缩小城乡收入差距。开展省级创业型城市创建活动，健全城乡就业服务体系，加强就业创业培训，推进劳动就业管理信息系统村村通工程。建成市级创业孵化基地10个，完成城乡职业技能培训25万人次、创业培训1万人次，新增城镇就业8万人、农村劳动力转移就业1万人，城镇零就业家庭保持动态清零。推进企业职工工资集体协商制度，构建和谐劳资关系。深入开展农村扶贫，完成10万低收入人口脱贫任务，100个经济薄弱村达到省定脱贫目标。

全面提升社会保障水平。进一步扩大社会保障覆盖面，提高社会保险市级统筹和保障水平。重点抓好非公经济组织从业者、灵活就业人员、进城务工农民的参保工作，职工五项保险参保人数平均新增1.8万人以上。城镇居民医疗、新农合、城乡居民养老保险参保率分别稳定在96%、98%、98%以上。继续提高企业退休人员养老金和城乡居民基础养老金。加快医保支付方式改革，进一步减轻参保群众负担。适时调整城乡低保和农村五保标准。完善征地补偿机制，逐步提高被征地农民基本生活保障标准。推进金保工程建设，发放社会保障卡40万张。进一步完善住房保障体系，推进共有产权式经济适用住房制度创新试点，扩大住房公积金制度覆盖面。全市新建各类保障性住房8000套，基本建成6200套，发放廉租住房补贴1650户。完善价格调节基金制度，健全惠民平价网络，新建平价商店20家。推进新一轮菜篮子工程，完成城区10个农贸市场改造升级。加快养老服务体系建设，实现公办示范性养老机构市、县全覆盖，建成居家养老服务中心269个，新增养老床位1000张。

加强和创新社会管理。深入推进社会管理创新工程，强化社会管理信息化建设，推行一委一居一站一办社区服务管理模式。依法做好村委会、居委会换届选举工作，切实保障群众民主权利。深化平安法治连云港建设，完善立体化社会治安防控体系，完成147个老小区安全防范建设任务。深入开展法制宣传教育，做好法律服务和法律援助工作，引导群众依法合理表达利益诉求。建立人民调解、行政调解、仲裁调解、司法调解联动格局，健全基层矛盾纠纷调处机制，落实信访工作责任制，努力把矛盾化解在基层。规范民间融资，防范化解金融风险。开展药品安全示范县区和食品安全示范乡镇创建，开工建设市食品药品检验检测中心。加强道路交通、工矿企业、建筑施工等重点领域的安全监管，做好校车安全保障工作。推进气象、防震等防灾减灾能力建设，健全突发事件应急管理体系。深化全国文明城市创建，加强社会主义核心价值体系和公民道德建设，大力弘扬雷锋车精神，努力营造和谐稳定的社会氛围。

八、切实推进政府作风新转变

严格落实中央和省市委关于改进工作作风、密切联系群众的有关规定，在提升行政效能上取得突破，努力建设人民满意的服务型政府。

改善政府服务。强化宗旨意识，坚持群众路线，力戒精神懈怠，克服能力不足，大力弘扬求真务实、埋头苦干作风，积极树立办文办会办事新风。巩固行政审批服务三集中四到位工作成果，完善项目代办、难题会办等有效机制，增强12345政府服务平台功能，积极帮助企业和群众解决实际困难，营造亲商扶商安商氛围。推进政府绩效管理，进一步提升行政效能，加大责任追究力度，着力治庸治懒治散，下大力气查处乱收费、乱罚款等行为，切实改善投资发展软环境。

坚持依法行政。严格按照法定权限和程序行使权力、履行职责，加强行政决策的合法性审查、风险评估和跟踪问效。加强公共资源交易市场建设，坚持重要事项和公共政策社会公示制度，推动行政权力网上公开透明运行纳入长效管理轨道。自觉接受人大法律监督和政协民主监督，认真办理人大代表建议和政协委员提案。完善四位一体政风行风热线媒体联动机制，积极构筑市长信箱、政务微博等沟通平台，主动接受社会和舆论监督，进一步增强政府工作透明度和公信力。

加强廉政建设。全面落实廉政建设责任制，健全廉政教育、权力监控、行政过错责任追究和纠风长效机制，强化对行政权力运行的监督和制约。推动电子监察系统向重点公共资源交易市场和重大政府投资项目等领域延伸，对国有资产管理、政府采购、土地出让、工程招投标、项目审批等加强行政监察和审计监督。坚持勤俭办事，大力推进节约型机关建设，严控三公经费支出。

各位代表，新一轮的发展时不我待，新一年的工作重任在肩。让我们在中共连云港市委的坚强领导下，昂扬斗志，满怀激情，开拓创新，苦干实干，强力推进全市科学发展、跨越发展，为全面建成更高水平小康社会而努力奋斗！
